# Bridging Nature and Engineering: Protein-Derived Materials for Bio-Inspired Applications

**DOI:** 10.3390/biomimetics9060373

**Published:** 2024-06-20

**Authors:** Taufiq Nawaz, Liping Gu, Jaimie Gibbons, Zhong Hu, Ruanbao Zhou

**Affiliations:** 1 Department of Biology and Microbiology, South Dakota State University, Brookings, SD 57007, USA; taufiq.nawaz@sdstate.edu; 2 Houdek, Brookings, SD 57006, USA; jaimie.gibbons@houdeknature.com; 3 Department of Mechanical Engineering, South Dakota State University, Brookings, SD 57007, USA; zhong.hu@sdstate.edu

**Keywords:** biomimetics, protein-based biomaterials, bio-inspired products, protein engineering, synthetic biology

## Abstract

The sophisticated, elegant protein-polymers designed by nature can serve as inspiration to redesign and biomanufacture protein-based materials using synthetic biology. Historically, petro-based polymeric materials have dominated industrial activities, consequently transforming our way of living. While this benefits humans, the fabrication and disposal of these materials causes environmental sustainability challenges. Fortunately, protein-based biopolymers can compete with and potentially surpass the performance of petro-based polymers because they can be biologically produced and degraded in an environmentally friendly fashion. This paper reviews four groups of protein-based polymers, including fibrous proteins (collagen, silk fibroin, fibrillin, and keratin), elastomeric proteins (elastin, resilin, and wheat glutenin), adhesive/matrix proteins (spongin and conchiolin), and cyanophycin. We discuss the connection between protein sequence, structure, function, and biomimetic applications. Protein engineering techniques, such as directed evolution and rational design, can be used to improve the functionality of natural protein-based materials. For example, the inclusion of specific protein domains, particularly those observed in structural proteins, such as silk and collagen, enables the creation of novel biomimetic materials with exceptional mechanical properties and adaptability. This review also discusses recent advancements in the production and application of new protein-based materials through the approach of synthetic biology combined biomimetics, providing insight for future research and development of cutting-edge bio-inspired products. Protein-based polymers that utilize nature’s designs as a base, then modified by advancements at the intersection of biology and engineering, may provide mankind with more sustainable products.

## 1. Introduction

The increase in demand for plastics has caused a major expansion in the production of plastic goods [1,2]. Every year, more than 100 million tons of plastic trash are disposed of globally, resulting in considerable environmental pollution due to the inherent resistance of plastic to natural disintegration [3,4,5,6]. The COVID-19 epidemic exacerbated the environmental problems associated with plastic wastes. Thus, it is important to reduce use of throwaway plastics [7].

Bio-sourced materials, possessing intrinsic biodegradability, are an attractive alternative to nondegradable plastic materials [8,9,10,11]. Natural materials possess the capacity to compete with synthetic polymers, particularly when they undergo appropriate processing.

Polylactic acid has gained significant attention as one alternative, due to its robust mechanical capabilities [12,13,14]. Unfortunately, degradation of polylactic acid requires a composting environment with suitable microbes to achieve degradation in a rapid timeframe [15]. Thus, scientists continue to focus on finding/developing polymers that have both exceptional mechanical qualities and sufficiently rapid degradation rates to be considered environmentally sustainable.

The protein-based polymers discussed in this study are predominantly sourced from natural products (Table 1). Proteins can be categorized according to structural and functional attributes, which determine their activities.

Given their lightweight properties and mechanical robustness, protein-based fibers demonstrate exceptional performance compared to typical artificial fibers (nylon and kevlar), particularly with regards to their unique biocompatibility, biodegradability, and sustainability [16,17,18,19]. This makes protein fibers especially favorable for biomedical uses, thereby broadening the spectrum commercial applications [20,21,22,23,24,25]. Alternative carbohydrate polymers, such as cellulose, hemicellulose, and chitosan, are described in other scholarly investigations [15,26,27].

**Table 1:** Structural characteristics of major nature-occurring proteins and their bio-inspired applications.

Endogenous Proteins	Applications	Structural Characteristics	Reference
Collagen	Membranous fibrils for the inhibition of cancer growth Geometric networks Beaded filaments	Tropocollagen Repetitive motif (Gly-X-Y) Alpha helical structure	[28,29,30,31,32]
Silk fibroin	Sutures and surgical threads Used as scaffold material for regeneration of skin, bone, and cartilage tissues Used in textile industry as silk scarves Used in packaging and as biodegradable plastics Used for optical device due to transparent and lightweight nature Edible films and coatings for food and pharmaceutical products Used as biomaterial for development of new material, and medical devices	Beta sheet secondary structure Hydrogen bonds	[24,33,34,35,36,37,38,39]
Fibrillins	Embryonic development Structural and organizational frameworks	Glycoprotein nature Calcium binding domains Cysteine rich domains Microfibril formation	[40,41,42,43,44,45,46,47]
Keratins	Used in shampoos, conditioners, and treatments for skincare products Used in cosmetics like nail polishes and foundations Used in textile industry as feathers and wool Used in bone grafts and dental membranes Used as natural fertilizer	Alpha-helix structure Coiled coil dimmers Intermediate filaments	[48,49,50,51,52]
Elastin	Sustain repetitive stretching Recover the deforming action after cessation Manage stress linked with pulsatile blood flow	Amorphous structure Elastin sheets and lamellae	[53,54,55,56,57,58,59,60,61,62,63]
Resilin	Used in robotics Used for the development of artificial joints, ligaments, and tendons Shock absorbing implants or prosthetic limbs Improve shock absorption and tire durability in tire manufacturing Create biodegradable and flexible plastics Used for tunable lenses and optical switches	High proline content Glycine rich domains Disordered structure Repetitive sequence	[64,65,66,67,68,69]
Wheat glutenin	Used for bioplastic and materials Bakery products Pasta production Processing meats Nutrition supplements	Polymerization Stress strain Gluten related disorders	[70,71,72,73,74,75,76,77,78,79,80,81,82,83]
Spongin	Tissue engineering scaffolds Controlled drug release Marine pollution monitoring Enzyme immobilization Artistic and craft projects	Fibrous and porous structure Cross linking Flexibility Biocompatibility Biosilica reinforcement Sustainability	[1,5,76,77,78,79,80,81,82,84,85,86,87,88]
Conchiolin	Jewelry and crafts Cosmetics and skincare Dental material Photonic and optical applications Textile and fabric	Layered structure Iridescent and pearlescent properties Organic matrix Lustrous appearance Biodegradability	[70,71,72,73,89,90,91,92,93,94,95,96,97,98,99,100,101]
Cyanophycin	Bioplastic production Nitrogen and carbon storage Bioprocessing Biodegradable additives	Multi-L-arginyl-poly (L-aspartic acid) Hydrophilic nature	[102,103,104,105,106,107,108,109,110,111,112,113,114]

This review is organized into eight sections. In Section 2, Section 3, Section 4 and Section 5, we review four different groups of protein-based polymers, including fibrous proteins (collagen, silk fibroin, fibrillins, and keratin), elastomeric proteins (elastin, resilin, and wheat glutenin), adhesive/matrix proteins (spongin and conchiolin), and cyanophycin. In Section 6, we review the techniques for application of protein-based materials in the post-treatments of endogenous and recombinant proteins, including the covalent crosslinking and hydrogel production. Section 7 describes novel approaches for the fabrication of protein fabrics. Section 8 gives perspectives.

## 2. Fibrous Proteins

Fibrous, globular, and membrane proteins are the three major classes of cellular proteins [115]. Fibrous proteins play a structural role by forming filamentous and sheet-like structures, which are typically inert and water-insoluble. Collagen, fibrin, keratin, and elastin are the four major fibrous protein families.

### 2.1. Collagen

Collagen is a fundamental structural component ubiquitously present in tissues [28]. It accounts for ~30% of the total protein content within the soma and serves as the principal constituent of the extracellular matrix (ECM). The primary function of ECM is to facilitate crucial structural reinforcement by augmenting tensile strength in various tissues and organs. Moreover, ECM is extremely important in providing tissue pliability, specifically for tendons [116]. Collagen is the most abundant fibrous protein in vertebrate connective tissues (tendon, cartilage, and bone) [117].

**Figure 1:**
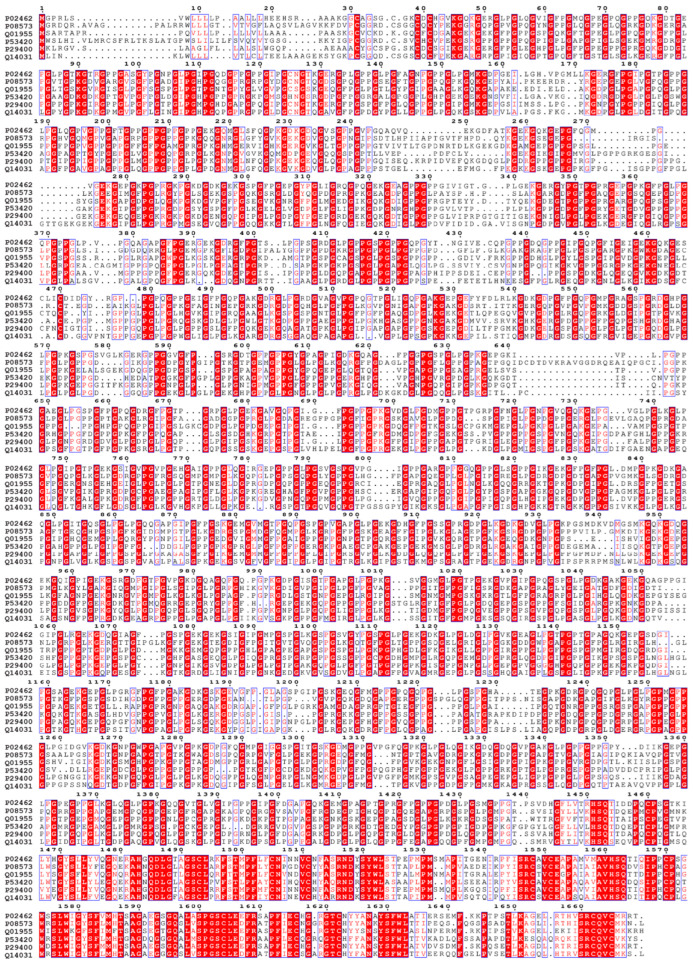
Multiple protein sequence alignment of Collagen type IV: Alpha 1–6 subunits shown the conserved G-X-Y repeated motifs.

In total, 29 distinct vertebrate collagens have been found, originating from a minimum of 42 unique genes [29,118]. Collagen has a unique structural attribute known as the amino acid triplet repeat, conserved Gly–X–Y motifs (Figure 1). “Gly” denotes the amino acid glycine, and the “X” and “Y” positions can accommodate any amino acid residue, however, proline and hydroxyproline are frequently found in these positions. Collagen types exhibit significant variations in their amino acid sequences, leading to distinct chemical and physical properties. Collagen types I to V are the most abundant and have been extensively studied for their biomedical applications [118]. Not all these collagens are part of the ECM.

Collagens are classified based on their α-chain composition and supramolecular structure. Fibrillar collagens possess significant quantities of continuous triple-helix structures (Gly-X-Y repeat motifs). On the other hand, α-chains have the potential to possess varying quantities of non-collagenous domains, hence introducing the possibility of interfering with the helical conformation.

**Figure 2:**
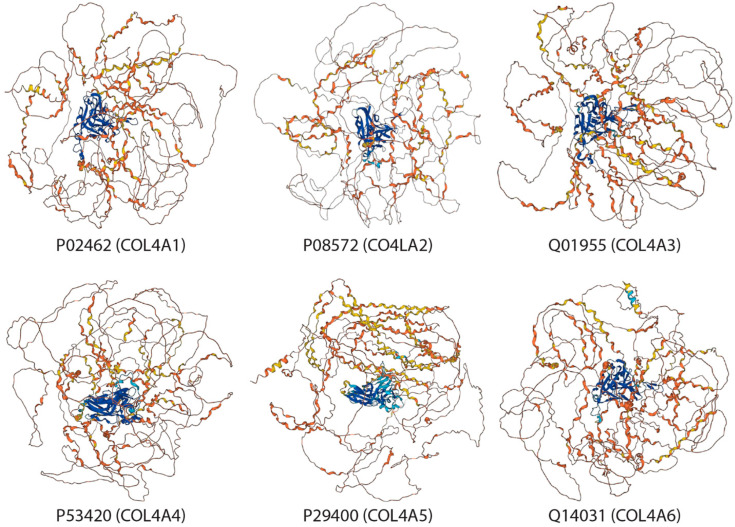
Collagen type IV Alpha 1–6 subunits’ structures predicted by AlphaFold Monomer v2.0 pipelne. The predicted local distance difference test (pLDDT) is a per-residue measure of local confidence. It is scaled from 0 to 100, with higher scores indicating higher confidence. Color legend:   Very high (pLDDT > 90);   High (90 > pLDDT > 70);   Low (70 > pLDDT > 50);   Very low (pLDDT < 50).

Collagen type IV consists of six genetically distinct isoforms (Alpha 1–6 subunits). Their sequence alignment is shown in Figure 1 and each subunit’s structure shown in Figure 2 as predicted by AlphaFold [119,120]. Type IV collagen serves as a key constituent of basement membranes.

Various forms of collagens are involved in the formation of diverse supramolecular architectures, such as geometric networks, membranous fibrils, and beaded filaments. Multiple kinds of collagen are present in many tissues, and they can co-distribute with diverse structural classes [30,31]. The presence of structural heterogeneity is associated with an increase in functionality. Various forms of collagens play significant roles in diverse biological processes and the inhibition of cancer growth [32].

### 2.2. Silk Fibroin

Several applications have been developed for silk due to its superior mechanical strength, which exceeds that of collagen [121]. Arthropod silk from arachnids, insects, and myriapods has potential to be an exceptional biomaterial. Silkworm domestication can be traced back more than 5000 years, as indicated by the oldest references originating from Chinese regions [122,123]. The *Bombyx mori* species has undergone significant domestication and cultivation in industrial farms owing to its capacity to generate superior silk that is well suited for textile applications [124]. The material exhibits several attributes, such as robustness, sheen, flexibility, elasticity, and the ability to establish chemical dye linkages. Moreover, its medical application for suturing wounds has also been practiced. The silk market exerts a significant influence on the worldwide textile economy, [75] with a total production of about 120,000 tons per annum of silk, primarily concentrated in Asia [125].

Due to the abundance of silk produced by *Bombyx mori* through sericulture, much research has been done on this subject. On the other hand, silk obtained from alternative arthropod sources, such as spiders, has not received the same degree of research interest. Arachnid species are deemed unsuitable for cultivation in controlled situations due to their predatory tendencies. Nevertheless, the silk produced by these organisms exhibits a distinctive amalgamation of robustness, high tensile strength, and excellent extensibility [34,73,74,126] surpassing that of other high-performance synthetic fibers. Due to their exceptional biocompatibility and biodegradability, the fibers are especially suitable for biological usage and exhibit extraordinary mechanical properties [23,36,127]. For instance, spider silk has been used in fishing lines in previous times, and more recently as microsutures [128,129]. In 2012, artist Simon Peers and entrepreneur Nicholas Godley created a cloak with a naturally golden hue, crafted from the silk of around 1.2 million *Nephila* spiders. The showing of this cloak in London had a significant role in establishing the silk’s status as a luxury item [130].

Researchers have been motivated by the remarkable mechanical attributes of natural spider silks, leading them to explore the creation of biomimetic protein fibers by recombining spider silk proteins [23,33]. Synthetic biology and recombinant DNA technology have facilitated the research and production of many variants of spider silk proteins (known as spidroins) [131,132]. Various spinning processes have been developed in the field of biological silk production [133,134,135,136,137,138]. The preservation of the hierarchical organization of recombinant spidroins exhibits limitations, which lead to missing functional domains. As a result, a considerable proportion of recombinant protein fibers demonstrate mechanical characteristics which are inferior when compared to innate spider silk. The examples in nature encompass mussel byssus [139,140], bagworm silks [141,142], sandcastle worm glue [143], and squid ring teeth [144,145]. The extensive range of patterns and shapes exhibited by these native proteins presents numerous possibilities for development of protein fibers possessing exceptional mechanical strength. Therefore, developing native structural proteins as biomimetic fibers is particularly attractive.

**Figure 3:**
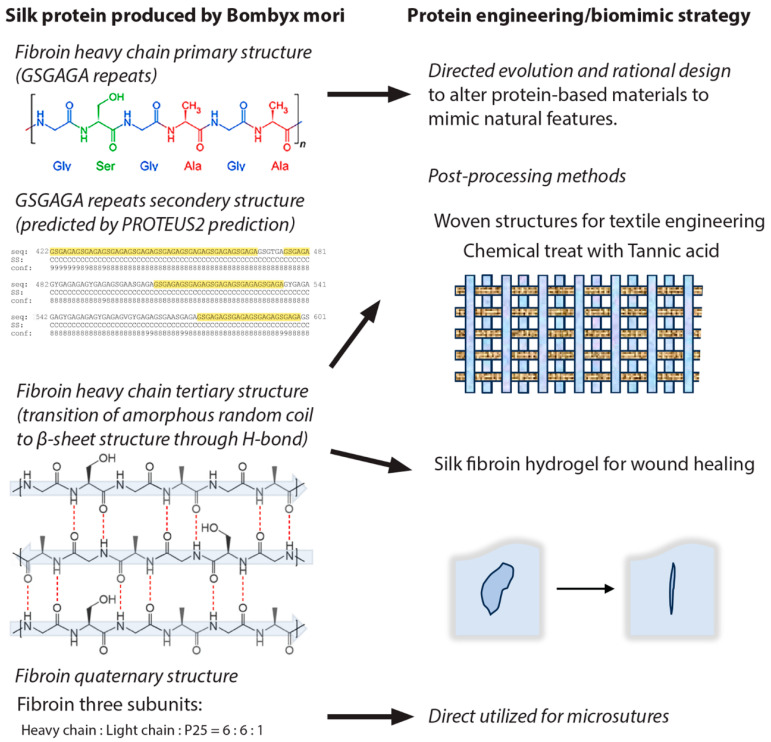
Schematic illustration of the naturally occurring protein-based biomaterial structures and their application, using silk fibroin as an example. (**Left**) Silk fibroin produced by the silkworm *Bombyx mori* consists of a heavy chain, a light chain, and a glycoprotein, P25. (**Right**) Examples of utilizing silk fibrin for various applications, including the silk fibroin hydrogel.

To better understand the mechanical properties of silk protein fibers, researchers have investigated the relationship among patterns of amino acids, structures, and mechanical attributes [35]. Fiber properties are affected by the arrangement of hydrophobic and hydrophilic domains within protein sequences that are commonly characterized by repetition. The presence of polyalanine within the hydrophobic zone is a contributing factor to high tensile strength, as indicated by previous studies [37,146]. Hydrophilic glycine/proline-rich areas facilitate hydrogen bonding between crystalline β-sheets. This interaction has a role in regulating fiber elasticity [147]. Silk fibers exhibit remarkable mechanical characteristics, rendering them exceedingly appropriate for the development of biomaterials employed in wound suturing, tissue regeneration, optical devices, biosensing systems, and drug administration endeavors (illustrated in Figure 3 [6,148,149]).

Silk protein fiber is composed of two components, fibroin and sericin [150,151]. The production of silk proteins occurs within epithelial cells that line certain glands of the silkworm. Subsequently, the proteins are discharged into the cavity of the gland and further converted into fibrous structures [152,153,154]. Glycine-X repeats make up the crystalline domains of silk fibroin, where X stands for the amino acids alanine, serine, threonine, and valine [155]. Fibroin is a dimer, consisting of a thin chain weighing approximately 26 kilodaltons and a thick chain weighing roughly 390 kilodaltons. There are identical numbers of both of these chains, and a single disulfide bond connects them [156]. The sericins are a class of sticky proteins that facilitate the binding and encapsulate of the fibroin fibers within silk. Sericins have a molecular weight range between 20 and 310 kDa [157,158] and are comprised of 44% glycine, 29% alanine, and 11% serine [159]. A bacterium bearing the genes from *B. mori* has been used to overexpress fibrion [101,160,161] and sericin proteins [162,163].

There are two different types of silk fibroin in its solid state. Silk I is the water-soluble initial state before crystallization and spinning. Silk I is then converted by exposure to heat, organic solvents or physical manipulation into silk II, which is the secondary structure also called a β-sheet [164,165]. The water solubility of the silk II structure is limited. However, it can be effectively dissolved in several chaotropic agents [34,166]. The β-sheets are stacked in anti-parallel alignment, with hydrogen bonds both inside and across chains and the influence of van der Waals force [156,167,168]. This structure provides high thermodynamic stability as verified using FTIR and circular dichroism measuring techniques [169].

Fibroin has found extensive application in medicinal products. The extraction of fibroin from the silkworm cocoon normally involves the removal of sericin, followed by a subsequent purification process [170]. There are numerous ways to extract and purify the protein from silk fibroin. The extraction of sericin, usually referred to as “degumming”, is frequently achieved using Na_2_CO_3_ boiling and autoclaving procedures. The degummed silk is next dissolved in a strong lithium bromide solution to separate the fibroin [171,172], or occasionally in a ternary solvent system comprising CaCl_2_, CH_3_CH_2_OH, and H_2_O [173,174]. Following the process of solvent evaporation, fibroin can undergo additional purification [159,175,176,177]. The potential of silk proteins for many biomedical uses is noteworthy owing to their capability to undergo easy processing in water or other solvents, resulting in the creation of gels, fibers, or sponges that can be chemically modified in a diverse manner. Furthermore, it is worth noting that these proteins exhibit notable biocompatibility, enzymatic degradability, and mechanical resilience as mentioned in previous studies [178,179].

Hydrogels can be formed via the sol–gel transition in reconstituted silk fibroin (SF) solutions, which can be triggered by the addition of acid, ions, or other supplementary substances [38,180,181,182,183,184]. Temperature, SF concentration, and pH are only a few examples of the variables that affect the gelation process. In general, gelation time exhibits a decreasing trend when the concentration of SF is increased, as well as when the temperature is elevated. Moreover, previous research has demonstrated a correlation between a reduction in pH and a corresponding decrease in the time required for gelation [182,184]. The hydrogel’s pore size diminishes while its mechanical strength and stiffness enhance as the concentrations of SF or gelation temperature increase [182]. The gelation period in silk fibroin can be controlled by adjusting the power output, duration of sonication, and concentration of the silk fibroin. This enables the manipulation of the gelation period, spanning from minutes to hours. Ultrasonication can enhance the creation of sheets. This, in turn, results in accelerated physical crosslinking and enhances the stability of gels. Conversely, subjecting SF hydrogels to vortexing results in a reduction in the gelation rate, hence prolonging the gelation process from a period of minutes to hours.

### 2.3. Fibrillins

Fibrillins are large, cysteine-rich glycoproteins, abundant in the ECM and distinguished by their large molecular weight of ~350 kilodaltons. Fibrillin consists of three members in humans: fibrillin-1, -2, and -3. They are the major structural components of microfibrils that are ubiquitously distributed in connective tissues. About 10% of completely grown elastic fibers are microfibrils, which are multiprotein fibrils. Fibrillin-1 is expressed throughout a human’s full lifespan, whereas fibrillin-2 and -3 are thought to be primarily present in early human development [40,41,42,185,186]. Fibrillin-2 is typically found in embryonic tissues but scarcely in adult skin, yet a marked increased expression was found in wound healing and sclerotic skin tissues [185].

All three fibrillins (≈350-kD) have similar modular organization that consists of 46 to 47 epidermal growth factor (EGF)-like domains (42 to 43 out of these are calcium-binding type cbEGF interspersed with seven 8-cysteine-containing TGF-β-binding (TB) modules found in LTBPs [43,187]. During the process of elastic fiber construction, microfibrils that are rich in fibrillin temporarily interact with various matrix proteins. The formation of fibrils with a thickness of around 10–12 nm is caused by the alignment of fibrillin monomers in a head-to-tail configuration. The fibrils have a beaded-string morphology characterized by the presence of beads separated by an average distance of roughly 56 nm. Additionally, it should be noted that the bead shoulder area of the fibrils consists of two symmetrical “arms” [44]. Calcium is of utmost importance in facilitating proper organization [45]. Microfibrils have traditionally been recognized as structural and organizational frameworks for elastin, as evidenced by their presence during embryonic development and their co-localization with tropoelastin [46]. The outer areas are the primary location for microfibrils in mature elastic fibers [47]. The functions of fibrillin microfibrils are distinct and autonomous, operating independently from their primary interaction with elastin. These functions entail enhancing the integrity of the ciliary zonules inside the ocular structure.

The maintenance of tissue structural integrity and flexibility is reliant upon the existence of collagen and fibrillins (elastic fibers). Tissues that possess collagen or elastic fibers frequently experience varying degrees and durations of deformations and provide mechanical resilience through unique mechanisms. The strength of collagen arises from its highly structured semi-crystalline molecular structure, which adopts a triple helical conformation. Nevertheless, the primary source of the material’s elasticity stems from the increased disorder of the polypeptide chains while in a relaxed condition and not subjected to stretching.

### 2.4. Keratins

Keratins are essential constituents of many biological structures, such as scales, hair, nails, feathers, horns, claws, hooves, and the outer layer of skin among vertebrates [188]. The silk fibroins produced by insects and spiders are often classified as keratins. Keratin has been used in wound healing, tissue engineering, and drug delivery [189]. The human genome has 54 functional annotated keratin genes (28 type I and 26 type II) [190]. The regulation of keratin production is affected by a diverse array of growth factors and cytokines, totaling more than 30 [191,192]. The formation of keratin fiber entails the development of a sturdy structure by employing covalent connections. Crosslinking can occur in two distinct manners: intermolecularly, which involves the bonding between separate polypeptide chains, and intramolecularly, which occurs inside a single polypeptide chain at different sites [49,193].

**Figure 4:**
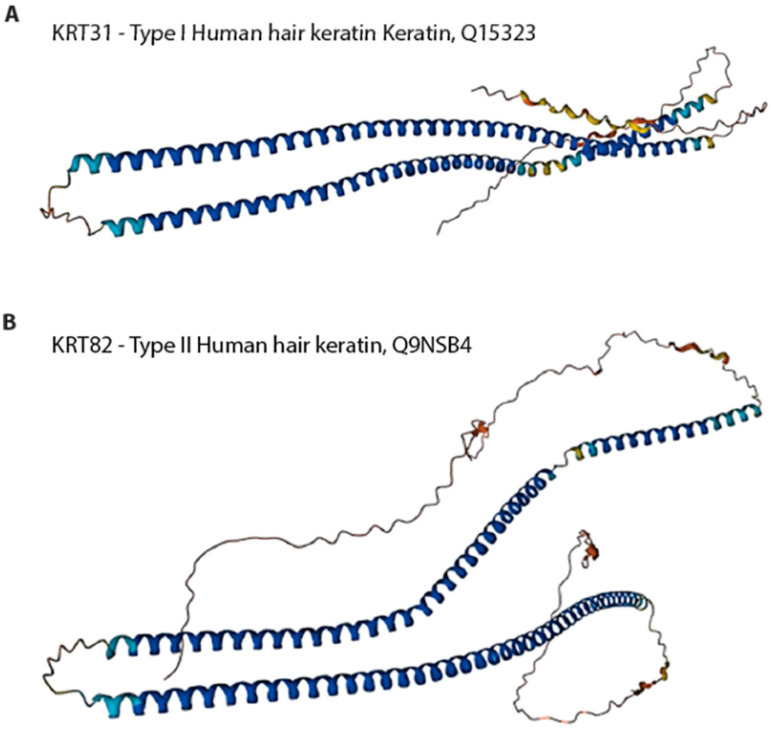
AlphaFold v2.0-predicted tertiary structure of type I keratin KRT 31 (**A**) and type II keratin IKRT 82 (**B**) from human hair. Gene ID: Q15323 (KRT31); Q9NSB4 (KRT82).The predicted local distance difference test (pLDDT) is a per-residue measure of local confidence. It is scaled from 0 to 100, with higher scores indicating higher confidence. Color legend:   Very high (pLDDT > 90);   High (90 > pLDDT > 70);   Low (70 > pLDDT > 50);   Very low (pLDDT < 50).

Keratins can be categorized into three discrete classes, specifically α-keratins, β-keratins, and γ-keratins. The α-keratins have an alpha-helical tertiary structure predicted by AlphaFold [117,118]. The predicted tertiary structures of type I keratin and type II keratin from human hair are shown in Figure 4. The average molecular mass typically ranges 60–80 kDa. The α-keratins possess the capacity for self-assembly, leading to the creation of elongated filamentous fibers that exhibit high tensile strength and great stretchability without experiencing breaking [189,194]. The β-keratins are primarily protective and form most of the cuticle. β-keratins do not form useful reconstituted structures.

The extraction of β-keratins is difficult [195]. The γ-keratins are globular, high in sulfur content, with a molecular mass of approximately 15 kDa. They demonstrate a compact, spherical conformation and are characterized by their heightened concentrations of cysteine, glycine, and tyrosine residues. The γ-keratins function as disulfide crosslinkers, holding the cortical superstructure together, which in turn enhances cohesion. The cross-linking mechanism described above [49,193] is responsible for the mechanical toughness and inertness seen in the cortical structure of hair and wool [196].

A variety of methodologies have emerged for the extraction of keratin. Both oxidative and reductive solvents have been employed in disrupting the disulfide crosslinks between cysteine residues, leading to the transformation of keratins into their non-crosslinked condition. Keratose is a protein combination that is acquired by employing oxidative solvents, facilitating the conversion of cysteine into cysteic acid. The protein combination obtained using reductive solvents, which maintain the cysteines in their native shape and facilitate the production of further crosslinks, is commonly known as kerateines. Keratose obtained using oxidative extraction methods, such as peracetic acid or hydrogen peroxide, demonstrates hygroscopic properties, water solubility, and the ability to form non-disulfide crosslinks, and susceptibility to hydrolytic breakdown in very acidic or alkaline environments. Biomaterials produced from keratose demonstrate a substantially accelerated disintegration rate when placed in an in vivo environment, often taking place within a time span that varies from a few days to a few weeks [197]. In contrast, biomaterials derived from kerateines using a reductive extraction method exhibit a prolonged duration of biocompatibility, as evidenced by their capacity to persist within the organism over a span of weeks to months. The reduced solubility of keratins in water-based solutions and their increased ability to withstand extreme pH conditions can be attributed to the oxidative coupling capability of cysteine groups, leading to the formation of additional crosslinks. Most frequently, sodium disulfite, 2-mercaptoethanol, or dithiothreitol are used in the reductive extraction technique [48,198,199,200,201,202].

The intrinsic self-organization of keratin solutions has been the subject of extensive research, encompassing both the small-scale and large-scale domains [203,204,205]. This phenomenon results in development of a three-dimensional fiber network, distinguished by a uniform configuration of fibers and porosity [206]. Films, sponges, and hydrogels are just a few examples of biomaterials made from keratin. In the early 1970s, attempts were made to create gels, films, and scaffolds using keratin protein solutions [207]. Blanchard was granted a patent for their use of a keratin hydrogel derivative of human hair to enhance the process of wound healing. To achieve the desired characteristics, the hair underwent an initial oxidation process using peracetic acid to synthesize hydrogels with these specific features. To cleave the disulfide bonds within the protein, the protein suspension was exposed to 60 °C for 4 h. Following this, the suspension was permitted to cool to ambient temperature, resulting in the breaking of disulfide bonds and the consequent synthesis of cysteine groups. This methodology improved protein solubility. Therefore, the development of a keratin-based hydrogel using only disulfide and hydrogen bonds seems to be a feasible strategy, eliminating the need for additional crosslinker chemicals.

### 2.5. The Development and Utilization of Fibrous Proteins

Fibrous proteins, which are alternatively referred to as scleroproteins, comprise a substantial category of proteins, alongside globular and membrane proteins. Collagen, elastin, and keratin are proteins that exhibit the ability to produce durable and flexible fibers, hence providing structural support for a wide range of tissues and organs [208,209,210]. Numerous animals, including silkworms, spiders, and insects, use fibrous proteins, such as silk fibroins found in cocoons, spidroins in spider webs, and resilins in insect tendons [208]. These proteins demonstrate a shared characteristic of repeating motifs in their sequences. Furthermore, the self-assembly of these proteins has an intrinsic attribute that facilitates a hierarchical self-organization, leading to the formation of fibrous structures characterized by well-defined mechanical properties [210]. The biocompatibility and mechanical resilience of these protein fibers make them particularly desirable in a range of medical and technical applications [211,212,213]. Furthermore, the engineering of recombinant protein variations enables the adjustment of multiple parameters, such as protein sequence, molecular sizes, and charge. The modifications have a significant impact on the ability to convert proteins into various configurations, such as particles, capsules, foams, nonwoven meshes, films, or hydrogels. The versatile structures of fibrous proteins have a wide range of applications in various fields such as medication delivery, tissue regeneration, optical systems, bioelectronics [210,213] and in biomaterial industries such as biofibers [214], bioplastics [215], biorubbers [216,217] used in textile innovations, environmental solutions, and biomedical applications [218].

## 3. Elastomeric Proteins

### 3.1. Elastin

Elastin is a stretchy protein that resembles a rubber band, which enables many tissues to sustain repetitive stretching or contracting and thereafter resume their shape upon the cessation of the deforming action [54]. Elastin is predominantly found within the ECM of vertebrates, except for lamprey and hagfish, which belong to the agnathans taxonomic group [56,219]. Owing to its extensive crosslinked structures, elastin is a long-lived protein that degrades slowly in healthy tissues and has a half-life of about 70 years [57,58]. The elastic fibers that are generated during embryonic development are required to endure repetitive stretching and recoiling over the course of an individual’s lifespan without experiencing permanent deformation or structural collapse [59].

Tropoelastin is the soluble precursor of elastin with a 60 kDa molecular mass, which can be characterized by the existence of two or three lysine domains. Domains of the KA type, exemplified by the sequence AAKAAKA, frequently demonstrate a recurring pattern in which lysine residues are surrounded by alanine residues. Lysine residues are also present inside KP-type domains, which are distinguished by a sequence that is abundant in proline and glycine, shown by the sequence PGAGVKPGKGP. The motifs of KA-type or KP-type form a helical structure that is left-handed and lacks intramolecular hydrogen bonding [60,61,62,63].

The flexible attributes of elastin are evident from its capacity to undergo substantial elastic deformation, as seen by its elastic modulus range of 0.3–1 MPa. Elastins exhibit a remarkable ability to undergo significant elongation with minimal application of stress. Elastin demonstrates the highest degree of linear elastic extension compared to all other documented biological substances, with a minimum extension threshold of 150% [220]. Moreover, this phenomenon exhibits a noteworthy degree of resilience, approximated at 90%, and exemplifies excellent longevity, as substantiated by the limited turnover throughout an individual’s lifespan [57,58]. Nevertheless, it should be noted that elastin exhibits a modest tensile strength of approximately 2 MPa.

It is crucial to underscore that the elasticity of elastin fibers is contingent upon their state of hydration. In the absence of moisture, elastin undergoes a transformation that renders it inflexible and susceptible to breakage [221]. The existence of water molecules within the polypeptide backbone has a significant impact in preserving the structural flexibility and disorder observed in the tropoelastin monomer, which is characterized by a state of high entropy. This phenomenon is widely acknowledged as a pivotal catalyst for the elastic recoil process [63,222,223]. Despite being in a disordered condition, tropoelastin monomers exhibit significant aggregation [63]. Proteins comprised of a significant percentage of non-polar amino acids are anticipated to exhibit an increased propensity for the establishment of a hydrophobic core, hence eliciting a repulsive effect towards water molecules. The core region exhibits a densely packed configuration of secondary structural elements, which serves as an efficient shield to safeguard the non-polar side. Elastin has distinctive properties due to its hydrophobic monomer, which consists predominantly of four non-polar amino acids (proline, glycine, valine, alanine), accounting for around 80% of its composition. The monomer exhibits notable disorder and flexibility when present in a solution [61,224,225,226].

The mobility of the elastin backbone remains preserved in both the aggregated condition [46] and fully grown cross-linked fibers [227]. The presence of structural instability in hydrophobic elastin domains and other elastomeric proteins can be ascribed to the significant proportion of elastic glycine and proline deposits. To meet the specified criteria, it is necessary for elastin to have a minimum proportion of 60% glycine and proline residues combined. Additionally, it is imperative that for every glycine residue, there are at least two proline residues [63]. The existence of these Gly and Pro residues hinders the development of elongated secondary structures. This phenomenon mostly arises due to the energetic disadvantage associated with the confinement of glycine residues within a certain structural arrangement. According to previous research [62], elastin sequences exhibiting hydrophobic properties and an average proline spacing exceeding eight are prone to β-sheet aggregation. The process of aggregation can result in the development of fibrils that resemble amyloid structures [228,229,230]. The presence of disorganized monomers and aggregates provides evidence for the entropic force that facilitates elastic recoil and the manifestation of rubber-like elasticity [231].

**Figure 5:**
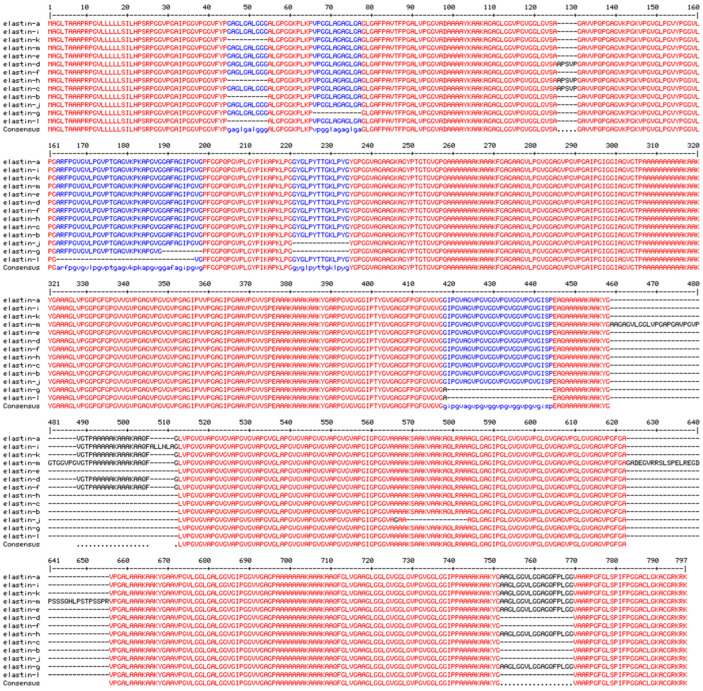
Sequence alignment for 13 human elastin isoforms (a-m).

**Figure 6:**
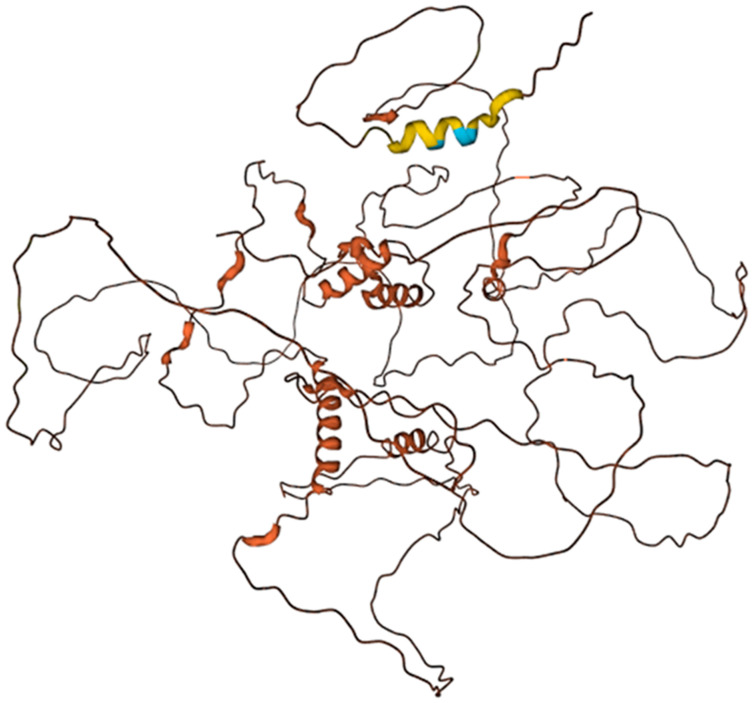
Human elastin K (E7EN65) structure predicted by the AlphaFold v2.0. The predicted local distance difference test (pLDDT) is a per-residue measure of local confidence. It is scaled from 0 to 100, with higher scores indicating higher confidence. Color legend   High (90 > pLDDT > 70);   Low (70 > pLDDT > 50);   Very low (pLDDT < 50).

The sequence alignment for human 13 elastin isoforms is shown in Figure 5. The human elastin K structure shown in Figure 6 was generated by the AlphaFold [119,120].

### 3.2. Resilin

Resilin is an elastomeric protein found in the exoskeletons of insects and arthropods. Its rubber-like elasticity is what allows insects to jump long distances, fly, and vocalize [64,65]. Resilin, one of the most flexible elastomeric proteins known, stores and releases energy due to its unique structure [232,233]. The estimated elasticity of resilin in dragonfly tendons is between 600 and 700 kilopascals (kPa). Resilin contains two unique amino acids (di- and tri-tyrosine), which give it a characteristic sapphire-blue fluorescence in ultraviolet light. Exceptionally resilient, resilin can elongate up to three times its initial length before reaching the point of breakage [234].

**Figure 7:**
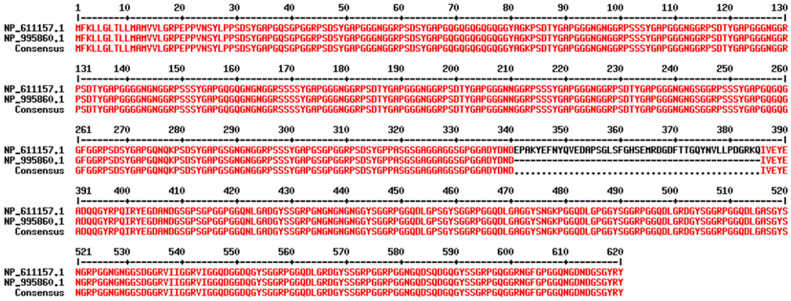
Sequence alignment for resilin isoform A (NP_611157.1, 620 aa) and isoform B (NP_995860.1, 575 aa) missing 45 aa (aa341–385) from *D. melanogaster*.

**Figure 8:**
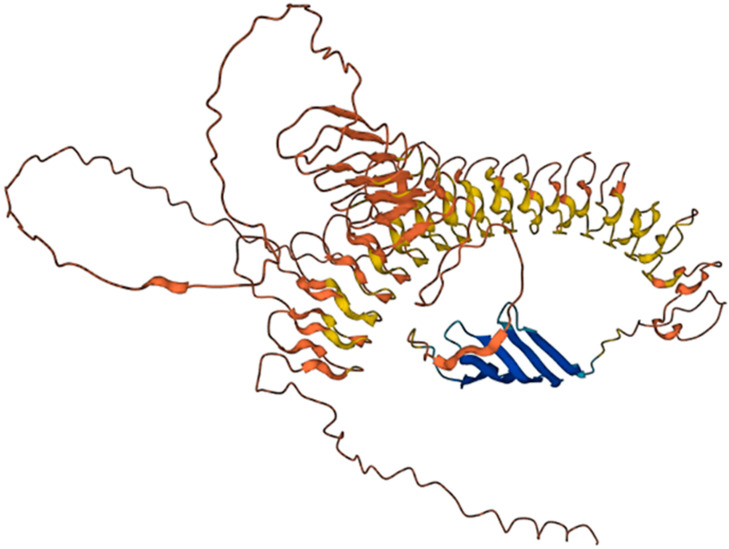
*D. melanogaster* resilin isoform A (NP_611157.1, 620 aa) structure predicted by AlphaFold v2.0. The predicted local distance difference test (pLDDT) is a per-residue measure of local confidence. It is scaled from 0 to 100, with higher scores indicating higher confidence. Color legend:   Very high (pLDDT > 90);   High (90 > pLDDT > 70);   Low (70 > pLDDT > 50);   Very low (pLDDT < 50).

A recently identified *Drosophila melanogaster* proresilin has glycine-rich repetitive sequences; the N- and C-terminal regions of the protein contain 18 repeats of a 15-residue sequence (SDTYGAPGGGNGGRP) and 11 repeats of a 13-residue sequence (GYSGGRPGGQDLG) [235], respectively. Conformational studies discovered the coexistence of two main features: folded β-turns and (quasi)extended structures (e.g., poly-L-proline II conformation). These features are common to other elastomeric proteins, suggesting a shared elasticity mechanism for resilin and other elastomeric proteins [235]. In addition to elasticity, resilin also lends itself well to fiber formations. A great tendency of resilin to aggregate in fibrous structures has been observed, particularly for the resilin-inspired polypeptide (PGGGN)10. This is encouraging for the development of resilin-based biomaterials to produce biocompatible medical devices, as well as high performing elastic materials. The sequences of resilin A and B from *D. melanogaster* are shown in Figure 7. The resilin A structure was predicted by AlphaFold and is shown in Figure 8.

### 3.3. Wheat Glutenin

**Figure 9:**
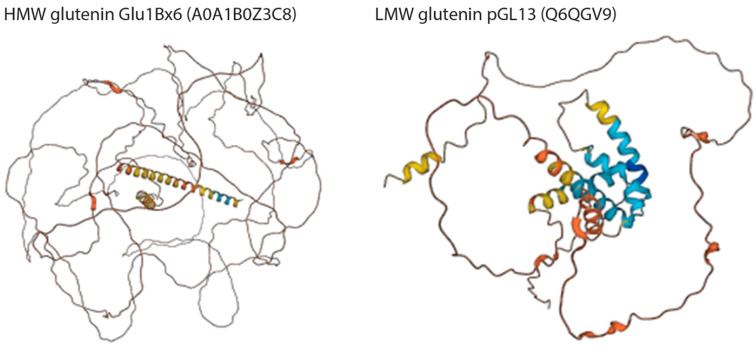
Wheat HMW and LMW glutenin structures predicted by AlphaFold v2.0. The predicted local distance difference test (pLDDT) is a per-residue measure of local confidence. It is scaled from 0 to 100, with higher scores indicating higher confidence. Color legend:   Very high (pLDDT > 90);   High (90 > pLDDT > 70);   Low (70 > pLDDT > 50);   Very low (pLDDT < 50).

Bioplastics can be made from proteins [236,237] from different sources. For example, wheat gluten [71,72,97] and casein show promising properties as a raw material for biodegradable polymers. Glutenin proteins consist of high-molecular-weight (HMW-GS) and low-molecular-weight (LMW-GS) glutenin subunits [72]. Wheat HMW and LMW glutenin structures predicted by the AlphaFold are shown in Figure 9. These GS are known to polymerize in the bread-making processes, forming one of the largest polymers in nature [73]. Wheat glutenin has been used in developing bioplastics [73,98].

Unlike wheat, N_2_-fixing cyanobacteria can use N_2_ gas as a sole nitrogen source to produce large quantities of proteins. Therefore, the Zhou laboratory at South Dakota State University is interested in engineering N_2_-fixing cyanobacteria to produce wheat glutenin: HMW-GS and LMW-GS to make biodegradable, proteinaceous plastics. To do so, the coding sequences for *hmw-GS* [238] and *lmw-GS* ((GenBank: AFU48612.1) will be codon-optimized and chemically synthesized at Integrated DNA Technologies (IDT). The chemically synthesized genes will be cloned into a cyanobacterial expression vector pZR1188 [239] to construct the cargo plasmid. Next, the cargo plasmid will be conjugatively transformed into *Anabaena* sp. PCC 7120 [240] for overproduction of the recombinant HMW-GS & LMW-GS proteins.

## 4. Adhesive/Extracellular Matrix Proteins

### 4.1. Spongin

Spongin is a modified type of collagen. It is believed to have emerged around 800–900 million years ago, during the emergence of multicellular animals. The poor solubility of natural spongin in acids and enzymatic treatments hinders its unequivocal identification as collagen, keratin, or a glycosylated derivative [84,241]. Spongin is responsible for the formation of 3D structured fibrous skeletons and remains an enigma of complex chemistry in water-insoluble structural proteins [241].

Spongin plays a crucial role as specialized templates for extreme biomimetics. It consists of biopolymers that exhibit high resistance to chemically harsh and thermally extreme environments. Renewable, chemically, and thermally durable biopolymers have not yet been used to fabricate novel biologically inspired composite materials. Such materials have potential applications in the most challenging environments of modern industry, including high-volume manufacturing. Recent research has shown that it is possible to create thermostable biopolymeric scaffolds using renewable structural biopolymers, such as proteinaceous spongin and aminopolysaccharide chitin. More precisely, the *Hippospongia communis* spongin scaffold was coated with TiO_2_ using an advanced biomimetic method. This novel biocomposite successfully eliminated C.I. Basic Blue 9 by means of adsorption and photocatalysis [85]. Furthermore, the synthesis of hematite (α-Fe_2_O_3_) was achieved using hydrothermal synthesis, employing the scaffold of *H. communis* as a template. The combination of hematite and spongin was proven to enhance the electrochemical properties of the capacitor electrode [86].

Due to their intricate 3D fibrous network structure and exceptional sorption properties, spongin scaffolds are a very suitable matrix for the immobilization of enzymes. *H. communis* spongin was studied as a model for immobilizing *Candida antarctica*’s lipase B (CALB). Surprisingly, even after being stored at 4 °C for 20 days, this biocatalytic system continued to show its efficacy. The immobilized lipase successfully facilitated the transformation of triglycerides into glycerol and fatty acid methyl esters, which holds great potential for the biofuel industry. Further studies were focused on the immobilization of enzymes found in the matrix of sponges [242], for example, the immobilized laccase enzyme from the *Trametes versicolor* mushroom substantially facilitated the degradation of bisphenols, which are dangerous compounds used in the manufacturing of polycarbonates [88]. In a separate study, a distinct biocomposite composed of *H. communis* spongin and iron phthalocyanine effectively eliminated several contaminants, including phenol, chlorophenol, fluorophenol, and bisphenol A [81]. In addition, researchers created a scaffold of a 3D carbonized spongin-Cu/Cu_2_O utilizing *H. communis*. This scaffold effectively catalyzed the conversion of the toxic compound 4-nitrophenol into 4-aminophenol [243]. The use of spongin-based scaffolds generated from the marine demospongiae *Haliclona sp.* has proven to be successful in the preconcentration and extraction of compounds such as ketamine [244] and fenitrothion [82].

### 4.2. Conchiolin

Conchiolin is a tough, insoluble protein secreted by mollusks, forming a major organic component of the shell with deposited calcium carbonate. Hydrolyzed conchiolin protein, obtained from pearl shells, is a frequently used cosmetic ingredient in hair and skin conditioning products [91,245]. Latire et al. analyzed shell extracts derived from the marine bivalve *Pecten maximus* [92]. These extracts were categorized as water soluble (WS), acid soluble (AS), and acid insoluble (AI) [93]. Following a 24 h period of incubation, AS significantly increased the metabolic activity of human fibroblasts. In addition, extracts from the mussels *Mytilus edulis* (AS and WS) and the oyster *Crassostrea gigas* (AS) were found to enhance metabolic activity and cell proliferation in primary human skin fibroblasts. These findings indicate the potential medical applications of these matrix proteins or their extracts, specifically for treating various skin conditions and promoting wound healing. Indeed, a study conducted on live rats with dorsal skin wounds shown that the gradual application of an ointment comprising powdered shells of *Megalobulimus lopesi* resulted in a reduction of the wound [89].

## 5. Cyanophycin

Cyanophycin is a natural biopolymer produced by a wide range of bacteria and is the third known naturally occurring amino-acid-based polymer, the other two being polyglutamate and polylysine [246]. Cyanophycin [247] is an N-rich reserve biopolymer in cyanobacteria consisting of equimolar amounts of aspartate and arginine residues, polymerized through non-ribosomal peptide synthesis. This polymerization is catalyzed by cyanophycin synthetase (CphA1), a unique amino acid polymerase [248,249]. Arginine is the most common side chain linked to the poly-aspartate backbone; however, lysine, ornithine, and citrulline have also been identified in cyanophycin produced in heterologous hosts [250].

Accumulation of Cyanophycin Granule Peptide (CGP) forms amorphous granular structures in the cytoplasm that are visible by light microscopy. Expressing CphA1 of *Anabaena* sp. PCC 7120 in *S. meliloti* led to a 2.8-fold increased accumulation of CGP, with the CGP content reaching 43.8% (*w*/*w*) of cell dry weight in recombinant cells of *S. meliloti* [251].

A single amino acid replacement in cyanobacterial P_II_ signalling protein (I86N) generated a P_II_ mutant (P_II_-I86N) strain *Synechocystis*-BW86 that accumulated up to 15.6% CGP by dry weight (DW), while CGP is barely detectable in the wild-type [252,253]. Phosphate or potassium starvation further increased the CGP content in strain BW86 up to 47.4% or 57.3% per DW, respectively [254].

Proposed applications of cyanophycin include bioplastic [115] and neutraceuticals [111]. Cyanophycin can form a poly-Asp polymer when arginine is cleaved from the poly-Asp backbone. Poly-Asp has potential uses in biodegradable plastics [105,115] or a possible substituent for polyacrylates [255,256]. Research on microbial production of cyanophycin has been well documented [102,256,257]. Many cyanobacteria are noted for accumulating CPG as a N-rich reserve polymer [258], but only N_2_-fixing cyanobacteria offer the opportunity to sustainably produce this N-rich biopolymer using sunlight and N_2_ gas as a sole nitrogen source. To genetically engineer N_2_-fixing cyanobacteria to produce a high yield of cyanophycin, Zhou’s lab will create a PII mutant of *Anabaena* sp. PCC 7120 using previously developed molecular genetic approaches [239,259,260,261,262,263,264,265].

### Cyanophycin-Based Biopolymers

Since aspartic acid is a component of the cyanophycin backbone, it is theoretically possible to hydrolyze cyanophycin using mild chemicals or enzymes to produce copolymers of poly-aspartic acid (PASP) with lower arginine concentration or even PASP homopolymer, a water-soluble and biodegradable polymer. PASP can be used as a dispersant for a range of organic and inorganic substances or as an antiscalant component in dishwashing and laundry detergents [112]. Furthermore, according to Mooibroek et al. [113], cyanophycin has the ability to be converted into a number of bulk compounds that contain nitrogen, including urea, 1,4-butanediol, and acrylonitrile. When cyanophycin is manufactured in plants, no extra facilities or specialized machinery are needed. Therefore, the product’s cost is very comparable to that of other polyamino acids, expanding the range of widely recognized uses for microbial cyanophycin [114].

Cyanophycin is also known as cyanophycin granule polypeptide. Many cyanobacteria synthesize cyanophycin as a short-term nitrogen reserve material. The cyanophycin synthetase enzyme catalyzes the polymerization reaction. Because purified cyanophycin may be chemically changed into a polymer with a lower arginine content, like poly-aspartic acid, it may be utilized as a biodegradable alternative to synthetic polyacrylates. This makes cyanophycin interesting for usage in biomedicine. However, bacterial production of cyanophycin on a large scale is severely impeded by the low yield and the highly complicated fermentation process, and the search is underway for new methods for large-scale production [110,113,257,266,267,268].

## 6. Protein Hydrogels

Proteins have four levels of structure: primary, secondary, tertiary, and quaternary. The primary structure is the sequence of amino acids in a polypeptide chain. The secondary structure is the α helix and/or the β sheet along with turn and loop components. The tertiary structure, also called 3D structure, is primarily due to interactions between the R groups of the amino acids that make up the polypeptide chain. Many proteins are composed of a single polypeptide chain and only have three levels of structure, while some proteins have multiple polypeptide chains (subunits) to make their quaternary structure. The sequence of amino acids in a protein is the primary factor that determines the bioactivity and nature of proteins and the four levels of structures [269]. The transition from the tertiary structure to the secondary structure is accompanied by conformational changes that result in an increased random coil structure. The increased random coil structure improves the flexibility of protein structure, making it more suitable for gelation [270,271].

Protein gelation is a phenomenon in which proteins undergo denaturation, resulting in their subsequent deposition inside a gel matrix, which is widely recognized and commonly applied for making protein hydrogels. Protein hydrogels can be generated through several methods, including physical, chemical, and enzymatic cross-linking procedures [272], which are facilitated by the intrinsic carboxyl and amino functional groups present in proteins. It has been shown that proteins containing cysteine residues demonstrate an enhanced capacity in the formation of hydrogels. The molecular structure of cysteine has a sulfhydryl (-SH) group, which accounts for its capacity to augment water retention and absorption capabilities. The swelling ratio of a material can be affected by several factors, including concentration of proteins, pH levels, and the presence of hydrophilic residues. Manipulation these parameters can be used to achieve the desired swelling characteristics in certain applications [273]. Yan et al. [274] altered the soy protein isolate and sugar beet pectin concentrations to change the swelling ratio of the hydrogel’s interpenetrating polymeric network. Joseph et al. [275] investigated the effects on the swelling ratio and adhesion characteristics through incorporating fibrin microparticles into hydrogels made of polyethylene glycol (PEG) and fibrinogen. Yan et al. [276] revealed that the hydrogel exhibited a lower degree of swelling in stomach fluid compared to intestinal fluid, which can be attributed to variations in pH levels.

Protein hydrogels are ideal materials for diverse medical applications, as they are biocompatible, biodegradable, accessible, and renewable. They can be redesigned and optimized through protein engineering. Protein-based hydrogels demonstrated favorable attributes, such as structural integrity, stability, strength, and a variety of unique properties like responsiveness to external stimuli and self-repair capabilities [277,278,279,280].

Polymeric hydrogels possess the capability to form a gel matrix through both covalent and non-covalent cross-linking methodologies. The incorporation of physical cross-linking methods in the production of protein-based hydrogels offers two significant advantages. Firstly, these procedures provide a simple and environmentally friendly operation, therefore distinguishing them from alternatives. Furthermore, they contribute to the retention of many of the protein’s intrinsic properties. Physical cross-linking plays a crucial role in augmenting the intermolecular contacts of macromolecular chains, hence promoting the production of polymeric biomaterial hydrogels [281]. Physically cross-linked hydrogels are formed through the synergistic effects of many weak intermolecular forces, including stacking, electrostatic interactions, hydrogen bonding, and physical entanglement between molecules [282]. A noteworthy advantage of physically cross-linked polybutylene hydrogels is their ability to undergo injection and self-healing at a temperature of 25 °C. Previous studies have demonstrated the efficacy of employing physical cross-linking techniques in maintaining the biocompatibility of proteins, thereby augmenting their potential utility within the realm of tissue engineering [282].

In the field of physical cross-linking, it is essential to initiate the denaturation of proteins, followed by their subsequent reassembly into their secondary structure. This process is crucial for the development of a gel matrix that exhibits a higher proportion of β-sheet composition [283]. Hu et al. employed ultrasound as a method to induce conformational unfolding for silk proteins, leading to an augmentation in the β-sheet content [284]. They later utilized hyaluronic acid to achieve physical cross-linking. Yan sought to achieve a similar objective by integrating hyaluronic acid into a silk protein solution. The researchers used a solvent exchange technique to produce a conformational arrangement typified by beta-sheets in the protein molecules. Consequently, the researchers achieved successful production of a hydrogel that demonstrated characteristics comparable to the silk protein [285]. Moreover, the concentration of proteins plays a significant role in the occurrence of physical cross-linking in this specific setting. It has been empirically shown that the protein demonstrates self-crosslinking as the concentration of the protein rises [217,286].

Polymeric biohybrid hydrogels can be synthesized by employing hydrogels with either a single network wrapping or multiple networks superposition. The mechanical characteristics of hydrogels composed of a single matrix are often limited. Tang et al. [287] utilized a protein misfolding methodology to produce a protein matrix. The protein was subjected to thermal exposure, leading to denaturation, aggregation, and subsequent gelation. Xu et al. [288] utilized a freeze–thawing method to generate a composite solution consisting of polyvinyl alcohol (PVA) and bovine serum albumin (BSA). In summary, the researchers synthesized the main hydrogel matrix as mentioned earlier. Following that, a secondary hydrogel matrix was produced through the process of physically cross-linking tannic acid with PVA and BSA, using hydrophobic interactions and hydrogen bonding. The study showed that the incorporation of both developed networks led to improved mechanical characteristics of the protein-based double-matrix hydrogel. The enhancement of feeble connections among lengthy macromolecular chains of polymers and proteins is encouraged by the elongated structure and the existence of electrostatic interactions or hydrogen bonds. The process of hydrogel production does not necessitate the complete denaturation of proteins. Without a doubt, the phenomenon of denaturation might be considered negative. The facilitation of the process can be effectively achieved through the structural transitions from tertiary to secondary conformation, or through the enhancement of β-sheet content. The occurrence of hydrogen bond production is responsible for a range of material characteristics, including thermoplasticity, self-healing ability, recyclability, customizable remodeling, and reusability. The presence of hydrogen bonds between residues and polymers can potentially aid the formation of dynamic bonds, leading to an increased likelihood of bond formation. The formation of hydrogen bonding by polar amino acids has been reported [289,290].

Numerous chemical cross-linking methodologies have been documented in scholarly publications, encompassing covalent cross-linking of protein chains and target residues, chemical coupling, and click reactions [291,292,293,294,295,296]. Prior research has indicated that the application of the cross-linking technique can enhance stability, regulate degradation rate, and exhibit notable mechanical properties in physiological conditions [297]. Moreover, the structural attributes of synthesized hydrogels can be modulated by the source of covalent linkages. The inclusion of polymers enhances the establishment of covalent linkages between proteins, leading to the development of hydrogels with unique properties. Hu et al. [284] examined the mechanism underlying the creation of covalent bonds. The chemical cross-linking of graphene, which had been modified with resilin-like proteins, was effectively achieved by the researchers. The technique of cross-linking resulted in significant improvements in several aspects, such as adhesion, elongation, and sensitivity. Wang et al. utilized a comparable modification methodology to facilitate the formation of C=C bonds by the integration of the vinyl group into gelatin. The aim was to produce methacrylate gelatin nanoparticles (MA-GNP) as the principal component for the crosslinking procedure [298]. Subsequently, acrylamide was added to MA-GNP to fabricate macromolecular microsphere composite hydrogels [6,298].

In a recent study by Su, chitosan was utilized as a vehicle for performing a chemical cross-linking technique, resulting in the formation of covalent imide linkages. Proteins with charge characteristics were utilized to fabricate hydrogels that have the ability to encapsulate diverse particles [299]. Huang et al. [217] conducted a significant study where they used a novel methodology to create hydrogels with rubber-like characteristics and specific mechanical properties. This involved integrating expanded silk networks into resilin blocks and employing photochemical cross-linking techniques. Through the manipulation of the silk to resilin ratio, the copolymers exhibited the capability to undergo self-organization, resulting in the formation of fibrous structures within a defined time frame. As a result, this event enabled the intentional formation of fibrils within copolymer solutions using supramolecular methods. The solutions were obtained using the light cross-linking approach implemented on supported hydrogels. Moreover, this served as a demonstration of the impact of the protein-to-polymer ratio on the mechanism of hydrogel formation. Furthermore, the integration of proteins into hydrogels can be utilized to alter and augment their characteristics. Wang et al. (2022) [300] have effectively developed a polymeric biomaterial hydrogel (PBH) that possesses noteworthy attributes such as anti-freezing capabilities, biocompatibility, and tunability. The achievement involved a two-step modification process. The first focus of the study involved the incorporation of anti-freezing proteins obtained from native fish species. Additionally, a chemical cross-linking technique was used to construct the desired polymeric hydrogel system [300].

The properties of hydrogels can be enhanced by integrating polymers with cross-linking or modifying qualities into the composition of polybutylene succinate-co-adipate structures. The polymers under consideration establish chemical interactions with proteins. Polymers have the capacity to serve as cross-linking agents enabling the modification of the amine and carboxyl termini of proteins. The aforementioned procedure results in the development of hydrogels that demonstrate significant resistance to fatigue, as supported by prior investigations [301,302]. Enzymes, serving as biocatalysts, offer notable benefits in facilitating and directing the hydrogel synthesis process. The characteristics indicated above encompass the performance of cross-linking reactions in mild reaction circumstances, demonstrating selectivity in terms of chemical, spatial, and stereochemical preferences, and decreasing the dependence on hazardous cross-linking agents [303]. In their latest study, Le Thi et al. [304] used enzymatic cross-linking as a supplementary strategy for cross-linking. The researchers developed a dual-enzymatic cross-linking approach using tyrosinase and horseradish peroxidase (HRP) in order to create a gelatin-based adhesive PBH [304]. The reactivity of gelatin towards nucleophilic molecules, such as amines or thiols, resulted in the creation of strong tissue adhesion. The reaction being examined involves the conversion of the phenol groups found in gelatin into o-quinone, assisted by the catalytic activity of tyrosinase. The utilization of a dual-enzymatic cross-linking approach has been shown to result in the production of hydrogels with greatly increased adhesive strength. Chirilaet et al. [305] conducted a study wherein silk fibroins were treated to self-crosslinking using the HRP enzyme and H_2_O_2_. The results of the study provided evidence supporting the benefits linked to the use of HRP in the process of cross-linking. The benefits that were noticed encompassed a noteworthy decrease in the gelation time, heightened flexibility, and improved compatibility with live cells. Hou et al. [306] performed an evaluation with the objective of developing a biofunctional PBH that is characterized by cost-effectiveness and simplicity. The researchers achieved this by exploiting gelatin and applying enzymatic cross-linking assisted by microbial transglutaminase (mTG) [306]. In the context of this framework, the application of mTG has led to the development of adhesive properties in gelatin microgels and the formation of a large hydrogel with pore sizes that promote cellular migration and proliferation.

## 7. Fabrication of Protein Fabrics

Multiple fiber-forming production techniques are routinely employed in many industries, such as melt spinning, dry spinning, wet spinning, and electrospinning [307,308,309,310,311,312,313,314]. These techniques utilize high-pressure solution extrusion to produce fibers, which are then subjected to other post-processing operations. The solidification of fibers in melt and dry spinning methods requires the application of cooling gases, but in wet spinning, precipitation is used to achieve the same objective [315]. After the fibers are formed, they undergo a sequence of procedures that involve collection, stretching, and alignment. These activities are facilitated using spindles and reels.

The electrospinning process is characterized by its deviation from conventional extrusion spinning techniques, as it relies on the application of an electric field within a voltage range of 5–20 kV, rather than solely depending on tensile force, to commence the formation of a polymer jet [316]. Electrospun fibers are produced with the use of an electric potential difference to a charged initial solution, with a grounded collector being employed. The solidification of the fibers is a result of the phenomenon of evaporation. The application of electrospinning in polymer fiber manufacturing is characterized by its versatility. However, it is important to acknowledge the existence of some constraints that restrict its effectiveness. These limits include a comparatively low production rate of 0.5 g per hour per spinneret as well as challenges in maintaining precise control over fiber diameter and orientation [315]. Previous studies have reported the impact of solvent conductivity on the trajectory of the polymer jet towards the collector substrate [317,318].

## 8. Perspectives

Most petroleum-based plastics are non-biodegradable, resulting in continuous accumulation in both terrestrial ecosystems and aquatic ecosystems. Plastic pollution causes severe environmental damage and threatens human health [1,5]. It is more pressing than ever to accelerate the development of biodegradable plastics to replace petroleum-based, non-degradable plastics, thereby reducing plastic pollution [319,320].

There are two major types of proteins in nature: fibrous proteins and globular proteins. Fibrous proteins offer a compelling prospect for making protein-based plastics to replace petroleum-based plastics. Fibrous proteins include keratin (hair, nails, feathers, horns), collagen (connective tissue), fibroin (silk), myosin (muscle), and novel filament-forming proteins (Fm7001 and All4981) recently identified in cyanobacteria [321]. The N_2_-fixing cyanobacteria are capable of photosynthetically producing high amounts of protein using only air (N_2_, CO_2_), mineralized H_2_O and sunlight. Cyanobacterial protein contents range from 46–71% in dry biomass [322,323], which is higher than conventional food protein sources, such as meat (43%), milk (26%), and soybean (37%) [322,323,324]. Although plant systems are now gaining acceptance as a platform for production of recombinant proteins, there is still resistance to commercial uptake. This is partly due to the relatively low yields achieved in plants, as well as being time-consuming and posing difficulties with downstream processing. Surprisingly, humans almost ignored the use of N_2_-fixing cyanobacteria, the “protein-making machinery” [322,323], having both the advantages of plant systems and prokaryotic systems, plus being capable of using N_2_ gas as sole nitrogen source to produce high yield of foreign proteins. Dinitrogen (N_2_ gas) resource is nearly 2000-fold more abundant (78%) than CO_2_ resources (0.04%) in the atmosphere and can be directly converted into amino acids by N_2_-fixing cyanobacteria, and fibrous proteins for making bioplastics can subsequently be synthesized through a synthetic biology approach.

Recently we also discovered in the genomic database of cyanobacteria that N_2_-fixing cyanobacteria may natively produce more than 11 super large proteins, the sizes ranging from 3883aa to 11342aa (Zhou R et al. unpublished data), much larger than the spider dragline silk proteins (3779aa). There has been no report of the use of N_2_-fixing cyanobacteria, a potentially commercially viable system, to produce full length spider silk proteins and any protein-based polymers mentioned above. Recently, research on photosynthetic production of recombinant proteins by cyanobacteria is underway [321,325,326,327]. Therefore, the N_2_-fixing cyanobacteria are the most attractive organisms chosen to overproduce N-rich polymers, the polymerized proteins for bioplastics. Using synthetic biology combined with machine-learning-guided protein engineering [328,329], we are very confident that we can genetically engineer N_2_-fixing cyanobacteria to produce all protein-based polymers mentioned in this review.

## 9. Conclusions

Synthetic biology combined with machine learning-guided protein engineering [328] has the great potential to create solutions for complex problems, subsequently revolutionizing our way of living, working, and understanding of nature. Synthetic biology designs and constructs new biological parts, devices, and systems that do not exist in nature. It is focused on engineering biology to produce commodities to benefit society, while biomimicry is about innovation inspired by nature to mimic nature’s genius to solve human problems. This review explored the promising future for protein-based biomaterials. The review targets protein-based polymers, focusing on their classification, application, structural arrangements, and integration into advanced patterning techniques. The classification of proteins based on their structural characteristics offers valuable insights into the diverse array of protein-based biomaterials, each possessing unique properties and potential applications.

The investigation and use of protein-based materials presents a promising domain within the realm of biomaterial research. This entails the innate natural design to generate innovative and sophisticated solutions. The qualities and activities of native proteins are largely determined by their various structural arrangements, which also provide the basis for improvement. Moreover, structural modifications present an intriguing prospect for creating protein-based novel materials with precision and control.

Generating patterns on photoresists by chemically modified proteins showed a significant potential of biomaterials in contemporary manufacturing methodologies. The use of a composite film derived from soy protein serves as an illustration of the wide array of protein sources that can be employed for the advancement of functional materials. The development of biodegradable wheat-glutenin-based polymers serving as environmentally friendly alternatives to petroleum-based plastics; the variety of applications for sponge in extreme biomimetics, bioinspired materials; and the promising future of conchiolin proteins in cosmetics and wound healing are just a few examples of the innovative and sustainable advancements being made in the field of protein-based materials.

A wide range of potential applications with protein-based materials is emerging from research into the interface of protein science, materials engineering, and advanced technologies. The entities possess unique structural configurations that, in conjunction with their ability to facilitate intentional modifications, play a central role in the advancement of patterned biomaterials. The application of a diverse array of proteins and their intricate structural properties enables researchers and engineers to propel the improvement of novel biomaterials, with potential implications across various domains including biotechnology and nanotechnology, among others. This review underscores the considerable potential of protein-based materials as a compelling avenue for scientific inquiry and technological advancement.

## Data Availability

Data sharing is not applicable. This is a review article that does not generate substantially new data.
